# The new X-ray/visible microscopy MAXWELL technique for fast three-dimensional nanoimaging with isotropic resolution

**DOI:** 10.1038/s41598-022-13377-w

**Published:** 2022-06-11

**Authors:** Yoshiki Kohmura, Shun-Min Yang, Hsiang-Hsin Chen, Hidekazu Takano, Chia-Ju Chang, Ya-Sian Wang, Tsung-Tse Lee, Ching-Yu Chiu, Kai-En Yang, Yu-Ting Chien, Huan-Ming Hu, Tzu-Ling Su, Cyril Petibois, Yi-Yun Chen, Cheng-Huan Hsu, Peilin Chen, Dueng-Yuan Hueng, Shean-Jen Chen, Chi Lin Yang, An-Lun Chin, Chian-Ming Low, Francis Chee Kuan Tan, Alvin Teo, Eng Soon Tok, Xu Xiang Cai, Hong-Ming Lin, John Boeckl, Anton P. Stampfl, Jumpei Yamada, Satoshi Matsuyama, Tetsuya Ishikawa, Giorgio Margaritondo, Ann-Shyn Chiang, Yeukuang Hwu

**Affiliations:** 1 RIKEN/SPring-8 Center, Hyogo, 679-5148 Japan; 2 Institute of Physics, Academia Sinica, Nankang, Taipei, 11529 Taiwan; 3 Research Center for Applied Sciences, Academia Sinica, Taipei, 11529 Taiwan; 4 Department of Surgery, School of Medicine, National Defense Medical Center, Taipei, Taiwan; 5 College of Photonics, National Yang Ming Chiao Tung University, Tainan, Taiwan; 6 Brain Research Center, National Tsing Hua University, Hsinchu, Taiwan; 7 Department of Pharmacology, Yong Loo Lin School of Medicine, National University of Singapore, Singapore, Singapore; 8 Department of Anaesthesia, Yong Loo Lin School of Medicine, National University of Singapore, Singapore, Singapore; 9 School of Chemical and Life Sciences, Nanyang Polytechnic, Singapore, Singapore; 10 ƐMaGIC-Lab, Department of Physics, National University of Singapore, Singapore, Singapore; 11 Mechanical and Materials Department, Tatung University, Taipei, Taiwan; 12 US Air Force Research Laboratory, Materials and Manufacturing Directorate, WPAFB, Fairborn, OH 43455 USA; 13 Australian Nuclear Science and Technology Organisation, Sydney, NSW 2234 Australia; 14 Department of Materials Physics, Graduate School of Engineering, Nagoya University, Furo-cho, Chikusa, Nagoya 464-8603 Japan; 15 Ecole Polytechnique Fédérale de Lausanne, 1015 Lausanne, Switzerland

**Keywords:** Imaging and sensing, Microscopy, Imaging techniques, Microscopy, Nanoparticles, Imaging techniques

## Abstract

Microscopy by Achromatic X-rays With Emission of Laminar Light (MAXWELL) is a new X-ray/visible technique with attractive characteristics including isotropic resolution in all directions, large-volume imaging and high throughput. An ultrathin, laminar X-ray beam produced by a Wolter type I mirror irradiates the sample stimulating the emission of visible light by scintillating nanoparticles, captured by an optical system. Three-dimensional (3D) images are obtained by scanning the specimen with respect to the laminar beam. We implemented and tested the technique with a high-brightness undulator at SPring-8, demonstrating its validity for a variety of specimens. This work was performed under the Synchrotrons for Neuroscience—an Asia–Pacific Strategic Enterprise (SYNAPSE) collaboration.

## Introduction

We implemented and tested a new microscopy technique called MAXWELL (Microscopy by Achromatic X-rays With Emission of Laminar Light) at the SPring-8 beamline 29XU^1^. The parallel use of X-rays and visible light achieves nanoscale resolution in all three-dimensions—avoiding the anisotropy of other microscopies—and is primarily conceived for large-volume biology specimens, including single-molecule imaging.

High resolution in the *z*-direction (perpendicular to the sample surface) is specifically important when using microscopy to identify microstructures, for example the neuron connections^2^. In standard visible microscopy, the *z*-resolution is limited by the elongated point spread function. This causes the aforementioned resolution anisotropy and makes it often difficult or impossible to extract accurate information on complex microstructures. The problem is even worse when super-resolution is achieved only in the *xy*-directions. With MAXWELL, the resolution anisotropy and the subsequent problems are avoided.

MAXWELL is implemented by irradiating the specimen with an X-ray beam shaped by a Wolter mirror as a very thin (< 65 nm) sheet (“nanoplane”). The X-rays stimulate the emission of visible light from the irradiated volume^3,4^ due to the presence of implanted scintillating nanoparticles (SciNPs)^5–7^. 3D imaging is realized by scanning the specimen through the nanoplane while sequentially capturing the visible emission.

## Results and discussion

**Figure 1:**
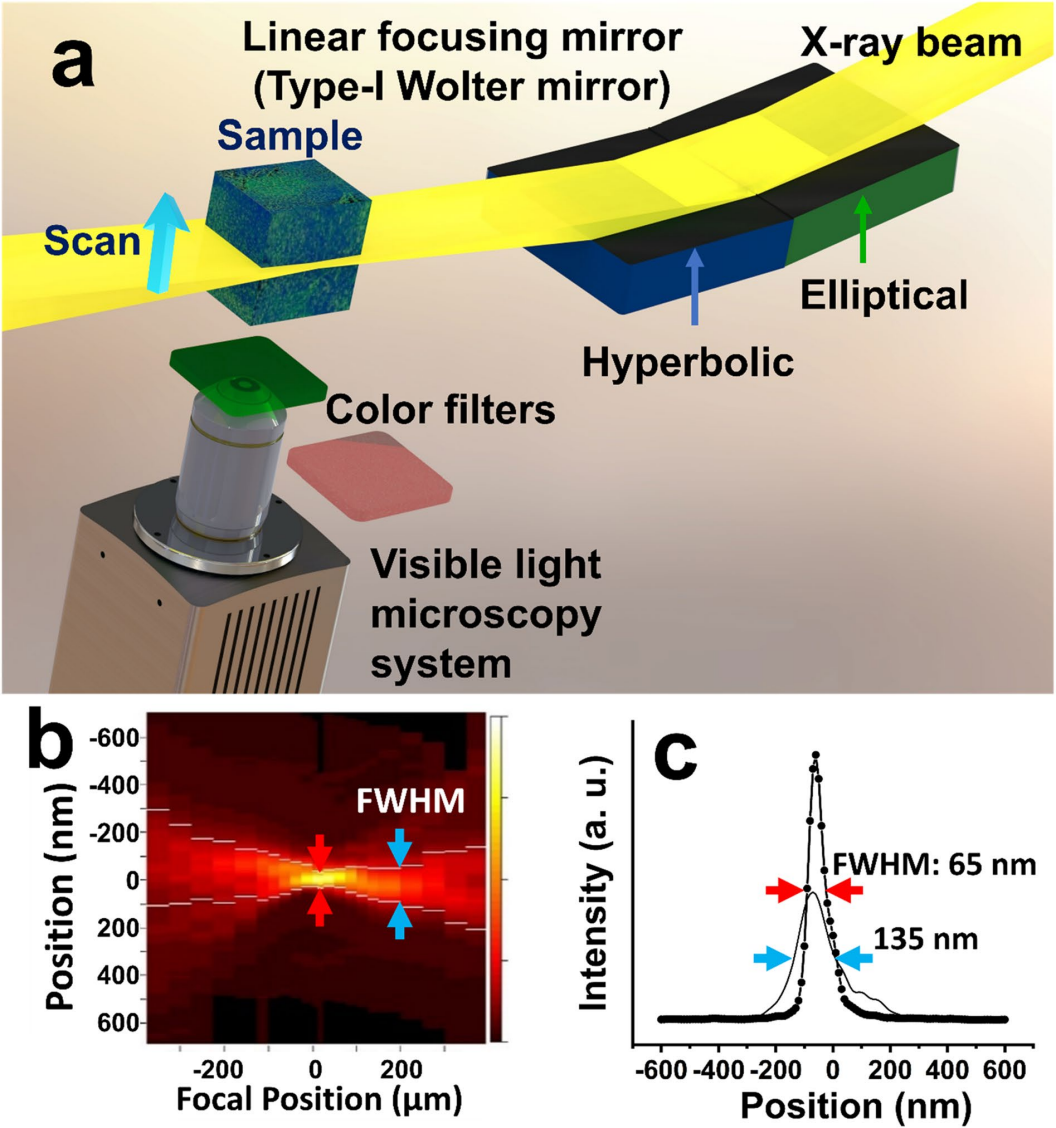
**(a**) Experimental setup of MAXWELL including a Wolter type-I linear focusing mirror that generates an X-ray nanoplane and a visible-light microscope system to detect the emission from X-ray-excited scintillating nanoparticles. (**b**) Knife edge intensity measurements performed at different positions along the optical axis. The measured thickness of the nanoplane was < 130 nm over the depth of focus of ± 175 µm. (**c**) A minimum FWHM size of 65 nm was observed at the focal spot.

Figure 1 shows the MAXWELL instrument and the resolution estimate. The high *z*-resolution of MAXWELL is possible because of the short wavelengths of X-rays. However, it requires a high-performance X-ray optical device^8–12^. Much progress was made in recent years so that X-rays can now be focused below10 nm^13–15^. Among all devices, the one-dimensional type-I Wolter mirror is a clear choice for MAXWELL.

Indeed, as other total-reflection mirrors it is almost achromatic and focuses X-rays over a relatively broad synchrotron-radiation spectral range. This corresponds to a high X-ray flux allowing high-speed imaging. The Wolter mirror produces a planar irradiation combining a nanoscale thickness (*z*-direction) and a large “in-focus” (*xy*-directions) area (> 400 µm × 400 µm). Figure 1b,c show the results of knife-edge intensity scans of the focused X-rays at different positions along the beam direction, referred to the focal point. This enables 2D full-field imaging and high-speed 3D imaging by fast scanning of large-volume (> 1 mm^3^) specimens. Such characteristics are difficult to achieve with other visible or X-ray focusing devices.

Furthermore, the Wolter mirror complements an elliptic-surface reflection with a hyperbolic reflection that eliminates the coma aberration^16^—enhancing the focusing stability, maintaining a fixed focusing size with no drifting and simplifying the alignment, which would be otherwise time consuming. To guarantee the required performances, state-of-the-art fabrication was used to produce ultrasmooth (< 0.2 nm RMS roughness and < 2 nm peak-to-valley figure error), aspherical reflecting surfaces^17^.

MAXWELL offers clear advantages with respect to other approaches for improving the *z*-resolution. For example, 4pi microscopy^18,19^and fluorescence lightsheet microscopy or selective plane illumination microscopy^20–24^ did not overcome the diffraction limit for *z*-resolution while adding constraints to the optical systems. Super-resolution in the *z*-direction was achieved using special fluorophores^25–27^ or by extracting the *z*-position of the fluorophores by quantifying their anisotropic aberration^28^. However, the long imaging time and the special specimen-labeling processes limit their use^29^.

On the contrary, MAXWELL achieves nanoscale *z*-resolution directly with X-ray focusing, without image processing. Since our SciNPs are much smaller than the X-ray beam thickness, the *z*-resolution is linked to the latter. Figure 1c shows a beam thickness of 65 nm. But this is not an absolute limit: considering the current resolution performance for X-ray focusing, further improvements are expected.

In addition to the thin X-ray nanoplane, small-size SciNPs are required to reach nanoscale *z*-resolution. For biological applications, small nanoparticles are also critical for precise administration to specific locations in tissues. And their visible emission should be ideally intense enough for single-particle detection. Tests of this capability, single particle imaging, using specimens with individual molecules are underway.

Note that the X-ray dose on the specimen when working at the highest resolution is ~ 10^15^ photons/mm^2^/s, which is quite high for biology tissues. There are margins for improving the apparatus performance and reduce this maximum dose. However, the feasibility of experiments on live specimens must still be assessed.

We extensively tested several 10 nm SciNP families, including NaGdF_4_:Eu and NaGdF_4_:Tb nanoparticles^30,31^ with red (~ 615 nm) or green (543 nm) emission, and CsBrPb_3_ nanoparticles with green emission (~ 530 nm)^32^. The TEM images of Fig. S1 show that the particle size is ~ 10 nm. The surface of these nanoparticles can be modified to conjugate linkers, such as streptavidin, to label specific macromolecules decorated with biotin. Such surface modifications, combined with tissue clearing^33,34^, allow 3D imaging deep into the specimen and single-molecule imaging for large samples.

Furthermore, with the relatively simple procedure^4^ of different dopings, SciNPs generate different scintillation colors (Fig. S2) and allowing multi-color imaging. We performed biocompatibility tests of these different SciNPs on live cells and found that there is only toxicity at very high concentration, 250 mg/mL (Fig. S3).

**Figure 2:**
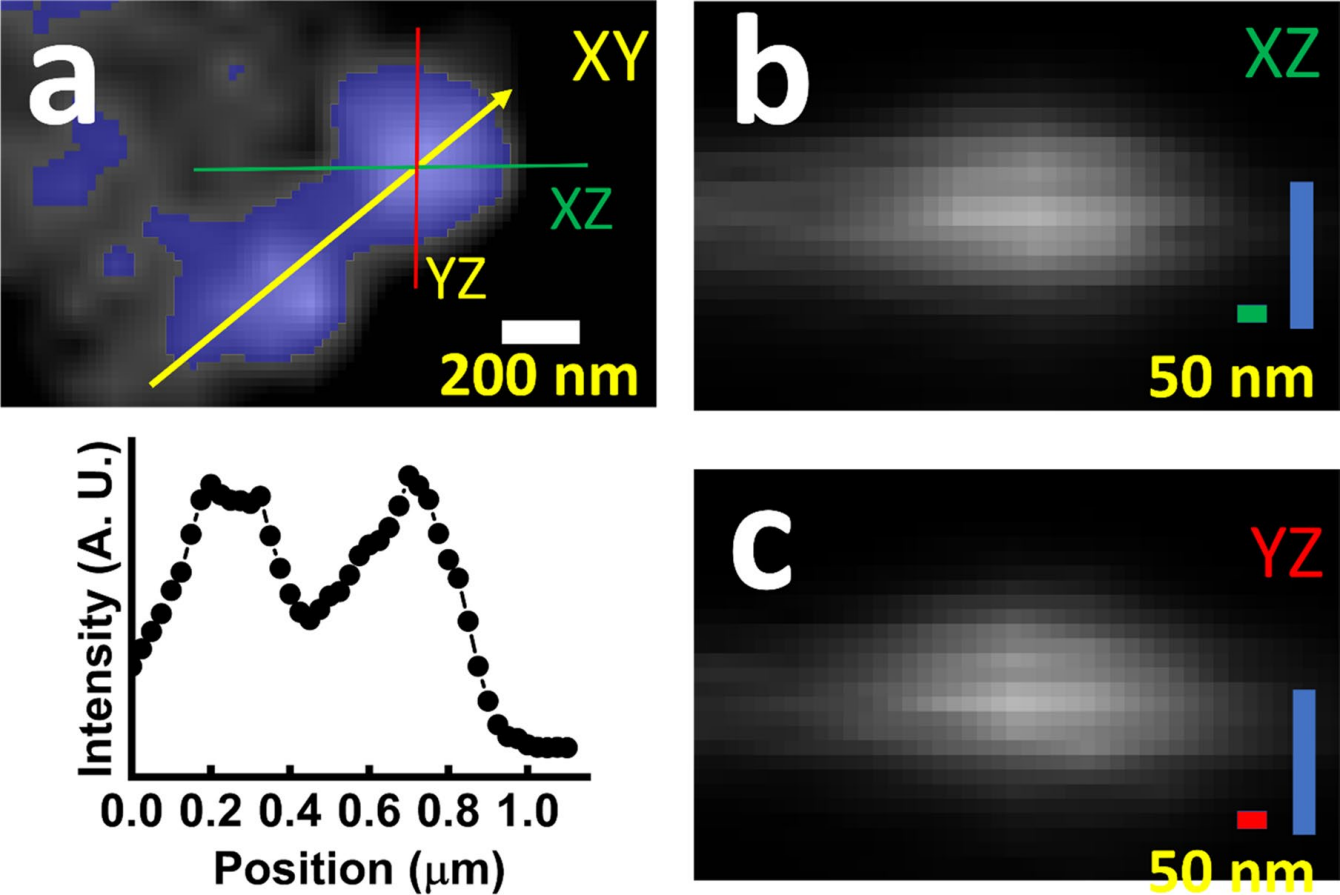
Assessment of the resolution in different directions from a 3D image with two CsBrPb_3_ SciNPs. (**a**) Image in the *xy* plane. An intensity profile along the yellow line is shown below. (**b,c**) The corresponding bright spot in the *xz* and *yz* planes.

Figure 2 shows images assessing the resolution in the *xy* and *z*-directions by finding the smallest light spots from the emission of SciNPs (CsBrPb_3_) dispersed on a test micropatterns. The images were taken with a 50× lens, 0.7 numerical aperture (NA). We see, in particular (Fig. 2a), two spots due to different particles and the corresponding intensity scan in the *xy* plane. From the distance between the brightest points and from the widths of the spots, we estimate a *xy*-resolution of ≈400 nm, consistent with the diffraction limit for visible light and with the NA. For the *z*-direction, Fig. 2b,c indicate a better resolution, of the order of at least 70 nm, consistent with the X-ray sheet width.

In the present situation, isotropic resolution can be achieved by increasing the X-ray sheet width and reducing the *z-*resolution. However, our final objective is to obtain isotropy by improving the *xy*-resolution, specifically using the “super-resolution” deconvolution^28,29^. Tests in that direction are underway.

**Figure 3:**
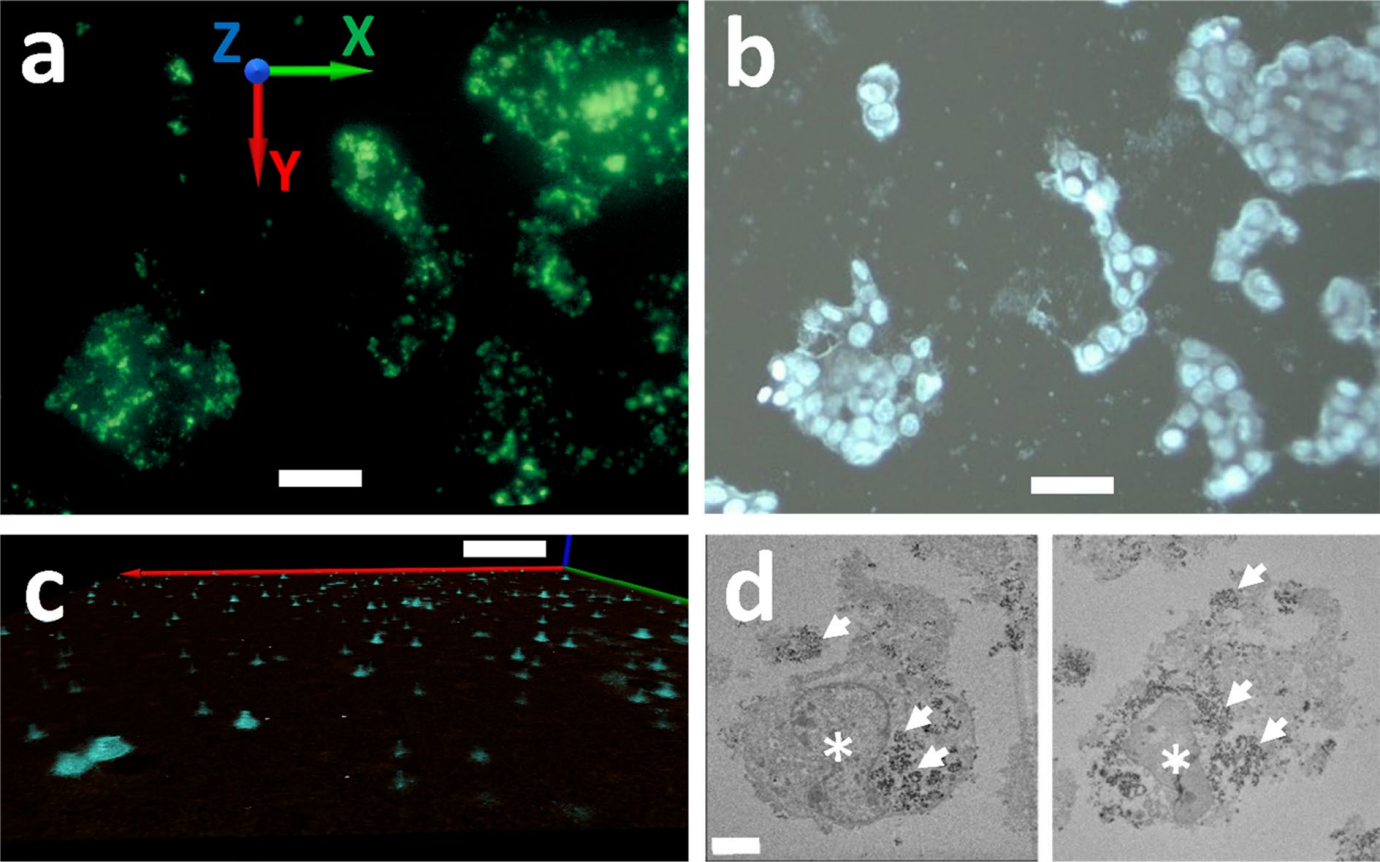
HCT 116 cells cocultured with SciNPs. (**a**) 3D reconstructed MAXWELL image. (**b**) image of the same sample taken with UV irradiation. Note that the color was presented from the 3D visualization process and does not represent the emission color of the SciNPs. (**c**) MAXWELL image of HCT 116 cells loaded with SciNPs. The *z-*direction has a scale 5 times larger than the *xy*-directions to show the 3D structure of the SciNPs. Note that particles appear distributed in the same plane because the cultured cells spread on the glass substrate, their distribution becoming very thin in the *z*-direction. (**d**) TEM images of an HCT 116 cell revealing that many NaGdF_4_:Eu (left) and NaGdF_4_:Tb (right) nanoparticles were internalized in the cytoplasm. The cell nuclei are marked by asterisks. Scale bars: (**a,b**) 50 µm; (**c**) 10 µm; (**d**) 5 µm.

Figure 3a shows one of the first MAXWELL field tests: a reconstructed image of a thin HCT116 cell specimen. The cells were cultured on a glass substrate with SciNPs (a mixture of NaGdF_4_:Eu and NaGdF_4_:Tb) internalized via the endocytosis process; the cell culture was then washed with PBS (phosphate buffer solution) to remove SciNPs from the culture media. The MAXWELL images show that the SciNPs only reside in cells, confirming preliminary tests with ultraviolet-excited fluorescence microscopy (Fig. 3b).

The bright spots in Fig. 3 are quite large. This is consistent with the cellular internalization of nanoparticles: they are aggregated by the endosomes and form large clusters of 0.1–2 µm size^35,36^. To show the 3D shape of these clusters, the *z*-scale of Fig. 3c is 5 times larger than for the *x* and *y* directions. Transmission electron microscopy (TEM) images (Fig. 3d) also confirm the nanoparticle clustering in the cell cytoplasm.

**Figure 4:**
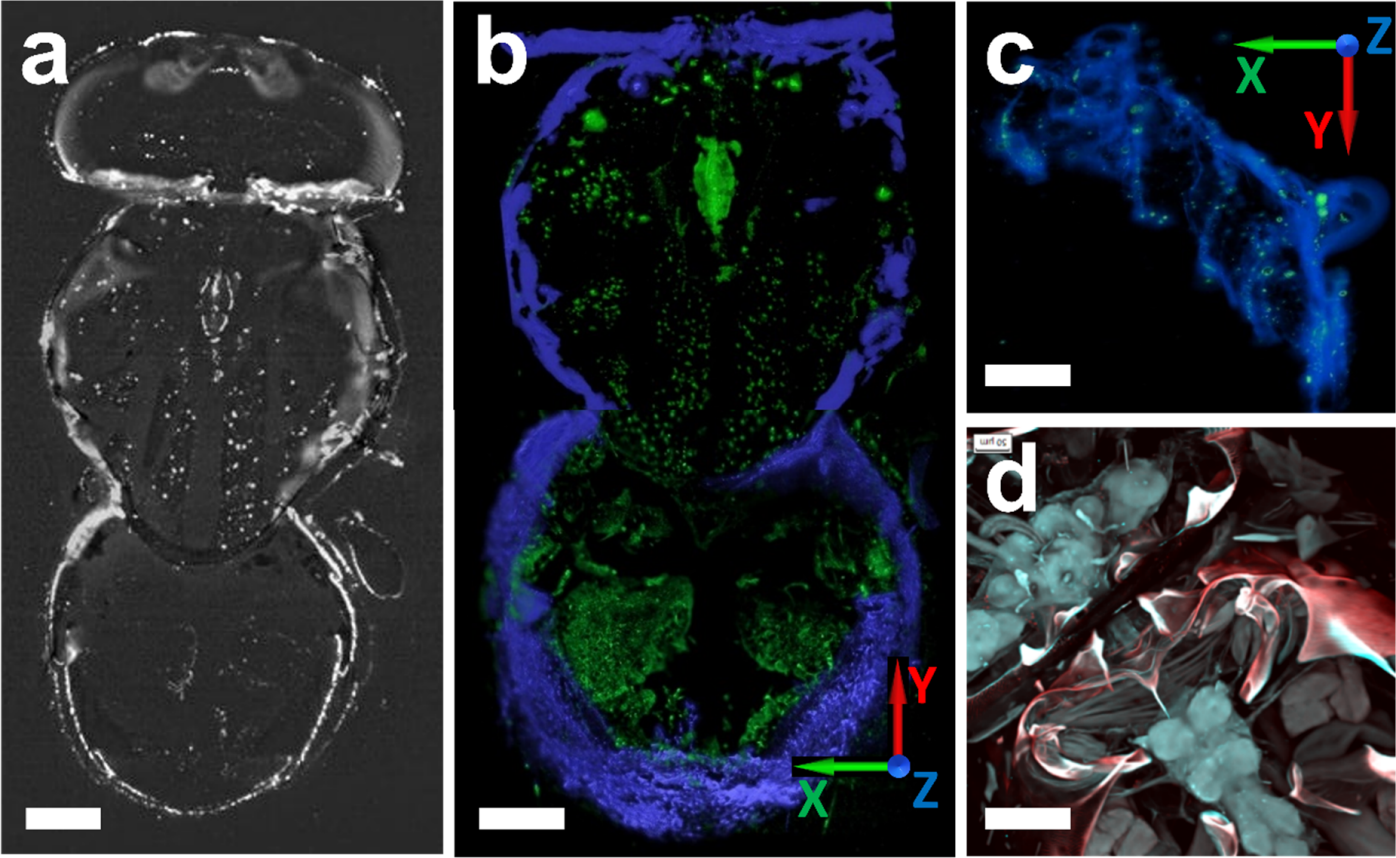
MAXWELL images show a drosophila fly with SciNPs (NaGdF_4_:Eu) administrated to delineate the tracheal and intestine features. The 3D image, (**b**), was constructed by stacking single slice images such as that of (**a**). Note that the head part is not reconstructed in (**b**). The skeleton (blue) is visible due to X-ray autofluorescence. The segmentation of autofluorescence and SciNPs are based on their intensities. (**c**) 3D reconstructed MAXWELL image of an entire Drosophila larva. (**d**) Confocal laser scanning microscopy of the same Drosophila specimen exhibits high contrast but the visibility of the SciNPs is much reduced. Scale bars: (**a**,** b**) and (**d**) 100 µm; (**c**) 50 µm.

Figure 4a,b show another MAXWELL test: a 3D reconstructed image of a drosophila fly with SciNPs deposited in the tracheal and digestive systems. The specimen thickness was ~ 200 µm. The upper (close to the objective lens) part of the inset was removed to better detect SciNPs in the thorax.

Figure 4c and the corresponding video clips (Supplementary Materials S02) show MAXWELL images of a thinner specimen, a Drosophila larva. The entire body was imaged without dissection. The X-ray-induced luminescence (blue) from the skeleton (labeled in blue Fig. 4b,c) provides a clear outline of the body^37^. The locations of the SciNPs (marked in green) delineate instead the tubular tracheal and digestive systems.

For comparison, Fig. 4d shows a visible-light confocal laser scanning microscopy image (FV-3000, Olympus Co., Tokyo) of the same fly specimen. This high-contrast 3D imaging could not detect the SciNPs, which were probably hidden by the much more intense fluorescence from other features.

Note that the signal-to-noise ratio (SNR) derived from MAXWELL images such as Fig. 4a reaches 11–12 decibels, much higher than the levels of confocal microscopy (Fig. 4d)—also thanks to the dark background. The high SNR would allow the detection of single SciNP, which is very stable in the X-ray irradiation without blinking or degradation of their emission.

Since the SciNPs formed in this case > 1 µm conglomerates, a focusing to a ~ 65 nm (Fig. 4c) or a relaxed ~ 170 nm (Fig. 4a,b) X-ray plane was used to irradiate the entire specimen. Note the uniform image quality over the *xy* planes, which is the result of the large depth of focus of the Wolter mirror. In this specific case, the X-ray nanoplane thickness of ~ 350 nm was maintained over a ~ (650 µm)^2^ area, sufficient for the required field of view in the *xy* directions. No deterioration of the resolution and contrast was detected over the entire volume. On the contrary, a visible lightsheet would produce only small-area “in-focus” images, because of the depth of focus of several µm.

**Figure 5:**
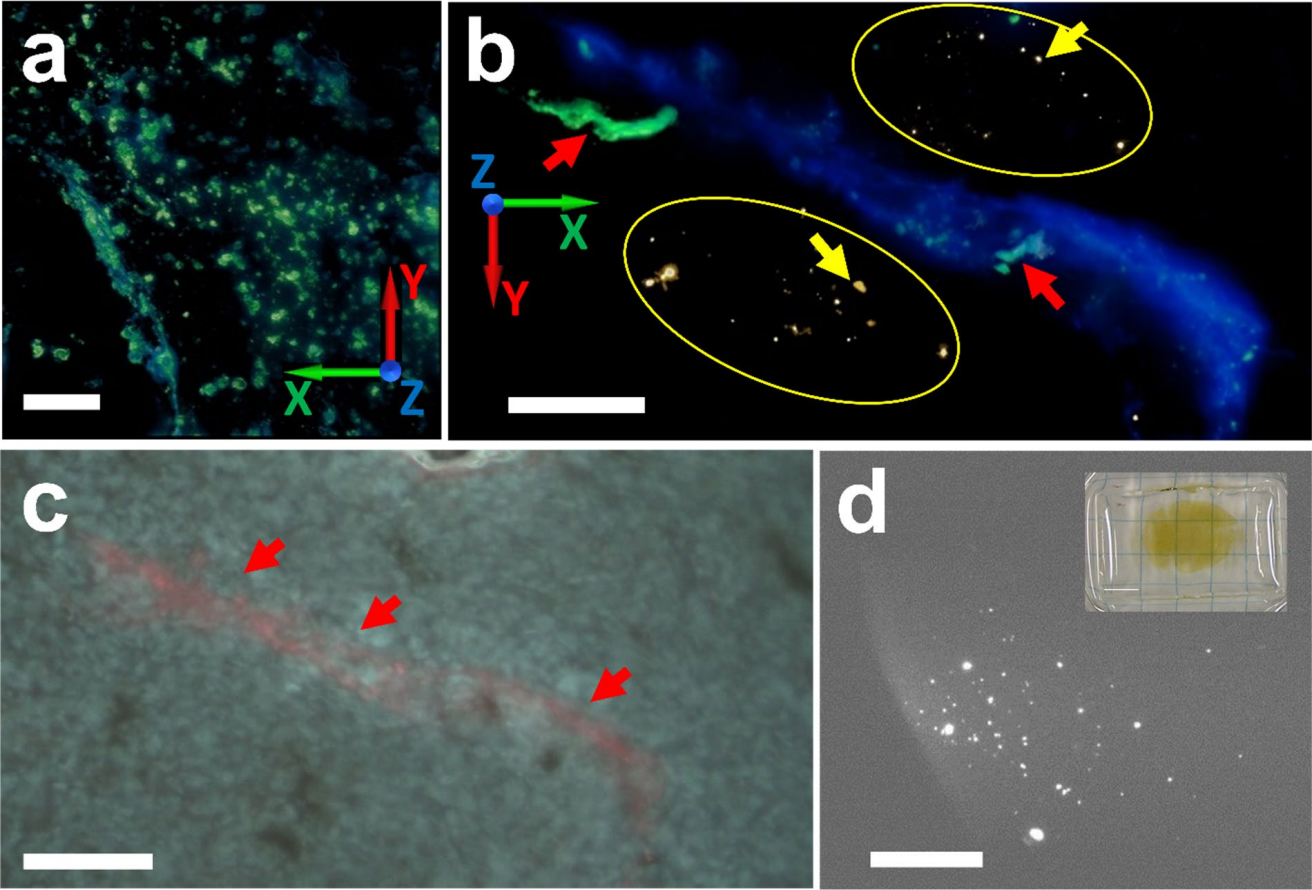
**(a**,**b**) MAXWELL images of mouse brain perfused with SciNPs (NaGdF_4_:Eu) to delineate the microvessels induced by tumor angiogenesis. SciNPs found outside the blood vessels, such as those marked by yellow arrows and circles, are attributed to the leakage through the fenestrations on the angiogenic microvessel walls. Red arrows mark microvessels with diameter ≤ 10 µm. (**c**) A micrograph obtained with UV illumination shows the red light emission of SciNPs aggregated in the microvessels. (**d**) Low-magnification MAXWELL images showing the aggregation of SciNPs in a region of a mouse brain. Inset: the entire brain imaged after tissue clearing. Scale bars: (**a**) 10 µm; (**b,c**) 50 µm; (**d**) 1 mm; inset: 5 mm.

Figure 5 shows a 3D reconstructed MAXWELL image of a slice of a mouse brain with glioma tumors. SciNPs were found, for example in Fig. 5b, accumulated in the tumor related microvessels via blood circulation^36^. A small amount of SciNPs was detected outside the microvessels, likely due to fenestration of the angiogenic microvessels.

Large-volume (> 1 mm^3^) imaging of biology specimen with MAXWELL is influenced by the self-absorption of emitted visible light by the specimen. This problem was alleviated with the *FocusClear* tissue clearing agent^38^ originally developed for visible microscopy. Figure 5d shows a MAXWELL image of a large region of cleared mouse brain tissue, without sectioning.

**Figure 6:**
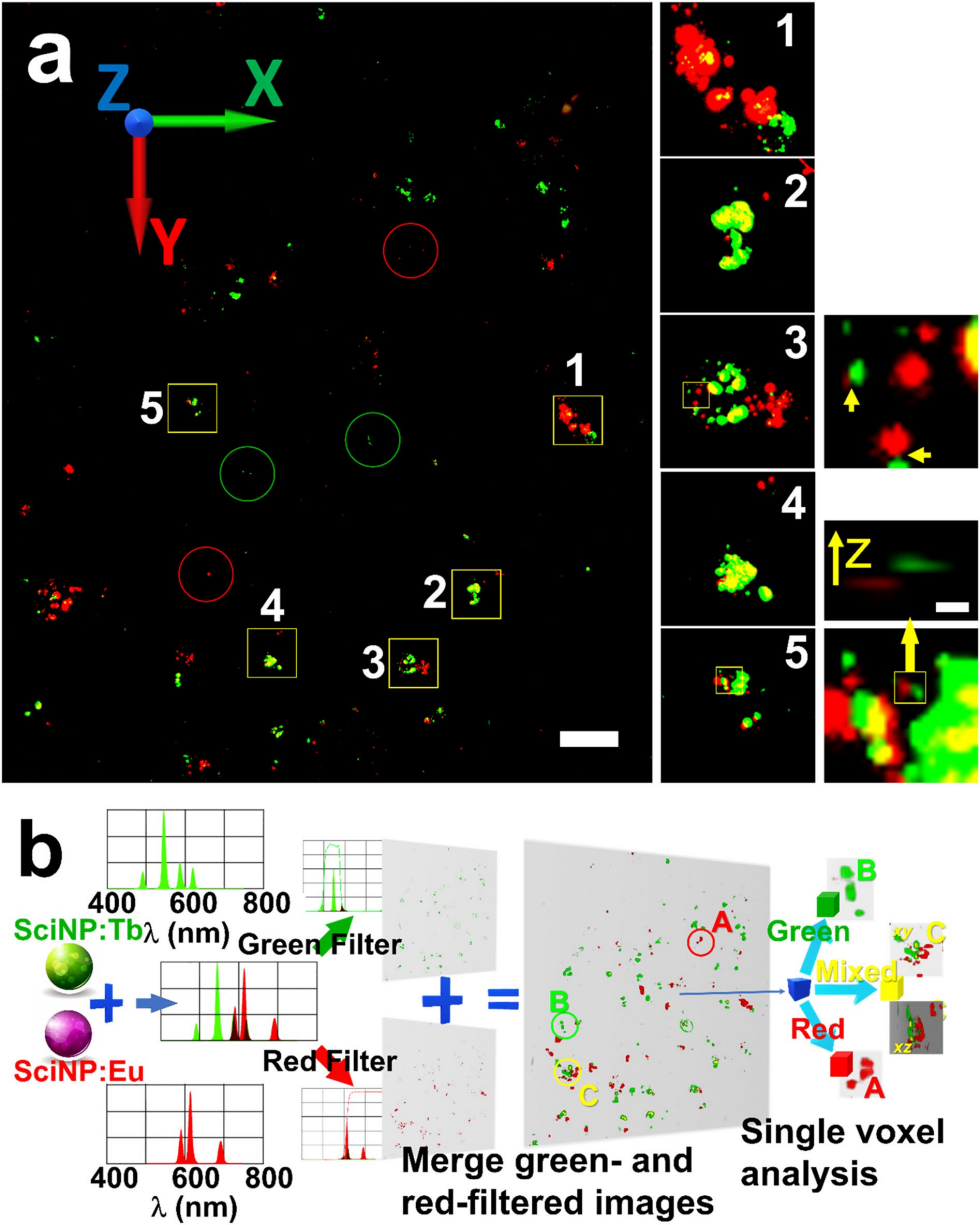
**(a**) Fused micrograph of two MAXWELL images taken with red or green filters, showing HCT-116 cells loaded with NaGdF_4_:Eu (red) and NaGdF_4_:Tb (green) SciNPs. Red and green circles mark those SciNPs emitting only red or green light. The yellow squares mark regions of mixed SciNPs (yellow). Zoomed-in images are shown on the right of (**a**). (**b**) Procedure to analyze and identify the color of each voxel of the merged 3D volume, **a**), with the emission spectra of red and green SciNPs. The voxels of strong green and red emission intensity are colored in yellow. Scale bar: (**a**) 50 µm; inset) 1 µm.

As previously mentioned, the SciNP emission spectra can be modified with different dopants while maintaining a small particle size and the surface properties^5,6,30^. Figure 6a shows a MAXWELL image of an HCT-116 cell specimen loaded with NaGdF_4_:Eu (red emitting) and NaGdF_4_:Tb (green emitting) SciNPs. Two separate MAXWELL 3D images obtained with red and green filters were merged into this picture.

Figure 6b illustrates the procedure to identify the emitting specific colors of each voxel of a light emitting region of interest using the emission spectra of the SciNPs. The 3D green-filtered and red-filtered images were aligned and merged. Each voxel exhibits both green and red emission. The red/green intensity ratio of each voxel was analyzed to identify its leading color—see for example those marked with red and green circles. Voxels with high red and green emission are marked in yellow: they are probably clusters of red and green SciNPs too close to separate. Note that the very high *z*-resolution, evident in the perpendicular views (the inset of 6a-1), facilitates the identification of nearby SciNPs of different colors (marked by a yellow square).

The key message of this last test is that SciNPs emitting different colors can enhance the results of MAXWELL using simple color filters. Other tests with different nanoparticles are underway. But we can already conclude that the combination of MAXWELL with nanoparticle manipulation and tissue clearing offers excellent flexibility in adapting the new microscopy to the needs of different experiments.

## Methods

The X-ray source for MAXWELL is the high-brightness SPring-8 BL29XU undulator equipped with a Si (111) double crystal monochromator^1^. A vertical slit with adjustable 10–30 µm width, at 45 m from the Wolter mirror, defines the virtual source.

The parameters of the Wolter mirror are the following: average total reflection angle of 5 mrad, half angular aperture of 1.5 × 10^–3^, substrate lengths of 232.2 mm and focal lengths of 229.6 mm. The mirror was fabricated with an ultra-smooth < 0.2 nm RMS surface roughness and an extremely small figure error (< 2 nm peak-to-valley)^16,39^ and coated with platinum. The diffraction-limited focus size at 10 keV X-ray is 41.3 nm. The angular range where coma aberration can be ignored is 80 µrad^39^.

The intensity distribution of the X-ray nanoplane and the corresponding focus size were measured using the dark-field knife-edge scanning method^40^. A platinum wire of 100 µm diameter was the knife edge. The intensity distributions along the beam direction, referred to the focal point, (e.g., Fig. 1b) were measured with 9 keV X-rays and a virtual-source size of 10 µm. The white bars indicating the FWHM (full width at half maximum) revealed a nanoplane thickness < 130 nm over a distance > 175 µm. The best focus size was 65 nm (FWHM).

With a virtual source width of 30 µm rather than 10 µm, the X-ray intensity increased 3 times. Even in this case, the focus size was < 90 nm for the entire X-ray energy range 9–11 keV.

The visible light produced by the X-ray irradiation is imaged by a microscope objective and an sCMOS camera (C14440-20UP, Hamamatsu Photonics K.K.). The camera has a 2304 × 2304 array of 6.5 µm pixels, and is used with microscope objectives with 1×, 20×  or 50× magnification. The sCMOS camera has low noise: read out noise is 0.7e and dark noise is 0.5 e/s, thanks to the Peltier cooling.

The visible light microscopy image of Fig. 4d was obtained with a confocal microscopy system (FV-3000, Olympus Co., Tokyo). The image was processed for deconvolution with the CellSens software (Olympus).

The specimen is mounted on a manipulation stage with 5° of motion. The X-ray nanoplane illuminates a fixed position coinciding with the focal plane of the visible objective lens. As mentioned, the 3D MAXWELL imaging is achieved by scanning the specimen in the *z*-direction with respect to the X-ray nanoplane.

The NaGdF_4_ core nanoparticles doped with Tb^3+^ or Eu^3+^ were synthesized as follows. Gd(CH_3_CO_2_)_3_·xH_2_O (0.85 mmol) and Tb(CH_3_CO_2_)_3_·xH_2_O (0.15 mmol) (or Eu(CH_3_CO_2_)_3_·xH_2_O) were dissolved in a combination of 6 ml of oleic acid and 15 ml of 1-octadecene. The mixture was heated to 130 °C under vacuum for 60 min and then cooled down to room temperature under nitrogen atmosphere. NaOH (5 mmol) and NH_4_F (4 mmol) were dissolved in 2.5 and 7.5 mL of methanol and stirred overnight at room temperature. The solution was then heated to 110 °C for 30 min under vacuum to remove residual methanol and moisture, purged with nitrogen three times, and finally heated to 300 °C for 120 min under nitrogen atmosphere. Finally, nanoparticles were precipitated with ethanol and washed three cycles with cyclohexane/ethanol (1:2, v/v), and the oleate-capped nanoparticles were dried.

Transmission electron microscopy (TEM, JEOL JEM-1200EX microscope, Japan) was used to analyze the nanoparticle size distribution. A drop of staining solution (2% phosphotungstic acid) was added before the SciNPs solution was deposited on copper grids or carbon films for TEM analysis.

HCT116 cells were grown in McCoy 5A (Modified) medium supplemented by 10% fetal bovine serum. The cells were incubated with 250 μg/ml of NaGdF_4_:Eu or NaGdF_4_:Tb SciNPs overnight. The cells were washed with PBS and then trypsinized and seeded with same amount of SciNPs loaded cells on coverslips to achieve 80% confluency. After 24 h incubation the cells were washed with PBS and mounted for imaging.

All procedures involving animals were approved by the Academia Sinica Institutional Animal Care and Utilization Committee (AS IACUC, approval number: 19-07-1326) and all methods were performed in accordance with the relevant guidelines and regulations. All methods are reported in accordance with ARRIVE guidelines (https://arriveguidelines.org) for the reporting of animal experiments. Eight weeks old C57BL/6JNarl mice were purchased from the National Laboratory Animal Center, Taiwan.

A total of 5 × 10^4^ GL261-GFP glioma cells in 2 μl PBS were inoculated via a 33 gauge needle into the basal ganglia of the right brain hemisphere of the mouse. The tumor was left to grow for 14 days before MAXWELL imaging. For the whole brain imaging as Fig. 4d, the mouse brain was treated with the *FocusClear* (ref. 38) tissue clearing agent for 7 days and removed from the medium before the measurement.

Before perfusion with SciNPs, the mouse was sacrificed by an overdose of Zoletil 50 (Virbac Laboratories, Carros, France, 1 mg/20 g bodyweight) administered by intraperitoneal injection. A catheter (PE-08, BB31695, ID 0.2 mm, OD 0.36 mm, Scientific Commodities, Inc.) was inserted into the right common carotid artery of the mouse and vessel is fixed with silk sutures. The tubing was secured with two knots around the CCA and then perfused with 2 ml heparinized (500 U/mL) NaGdF_4_:Eu SciNPs (10 mg/mL in 5% glucose buffer). The brain tissue specimens were immersed in 4% paraformaldehyde for 24 h. After fixation, the tissues were washed by PBS three times and sliced to 100 μm thickness by a vibratome (VT1200s, Leica Biosystems, Buffalo Grove, IL, USA). The slices were embedded in *EverBrite™* Hardset Mounting Medium with DAPI (Biotium, Fremont, CA, USA).

## Supplementary Information

Supplementary Information.

Supplementary Video 1.
