# Supplementary material for: The new X-ray/visible microscopy MAXWELL technique for fast three-dimensional nanoimaging with isotropic resolution
Source: Sci Rep. 2022 Jun 11;12:9668. doi: 10.1038/s41598-022-13377-w (PMC9188605; doi:10.1038/s41598-022-13377-w)
Supplement: Supplementary file 1 — Supplementary Information. [file 41598_2022_13377_MOESM1_ESM.docx]

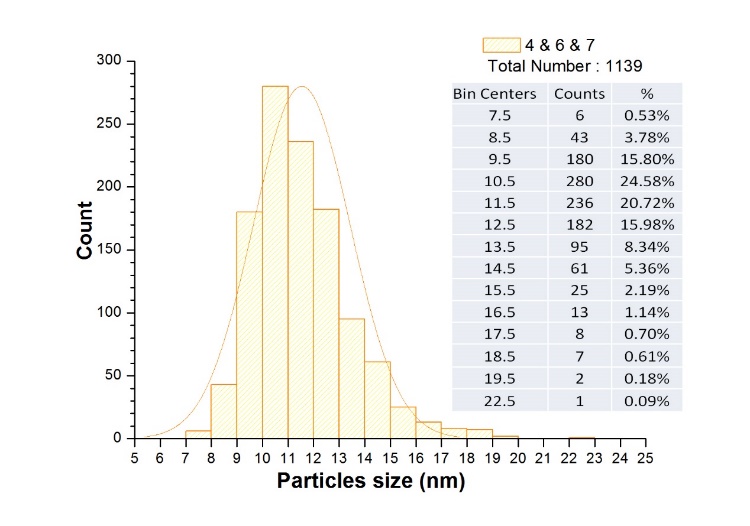


**Particle size (nm)**

**Counts**

**300**

**200**

**100**

**0**

**6 8 10 12 14 16 18 20**

**50 nm**

**NaGdF_4_:Tb**

**d**

**c**


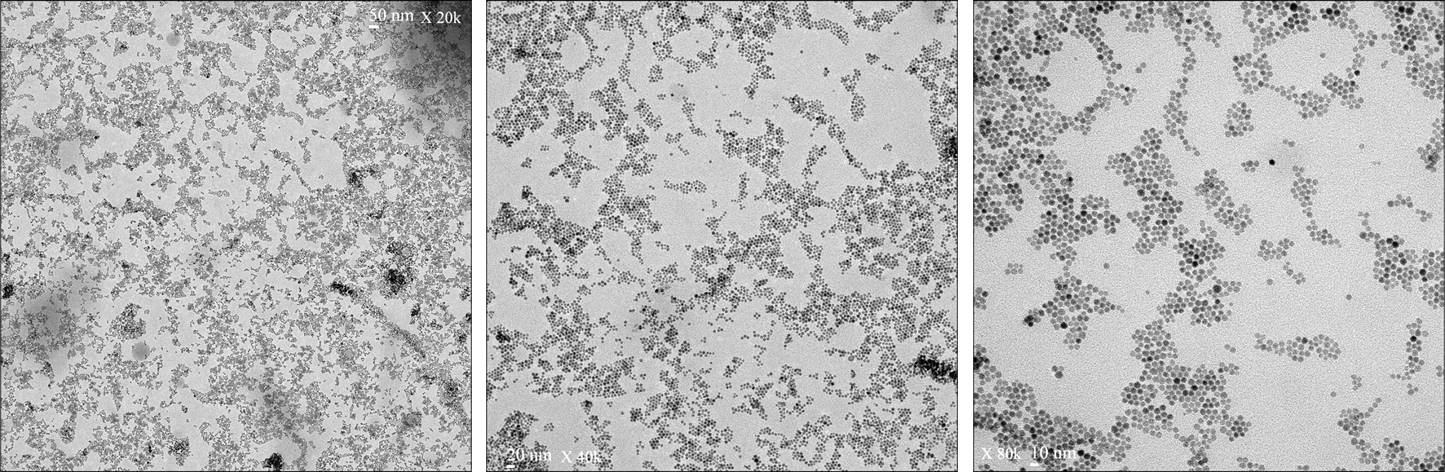


**NaGdF_4_:Tb**

**100 nm**

**b**


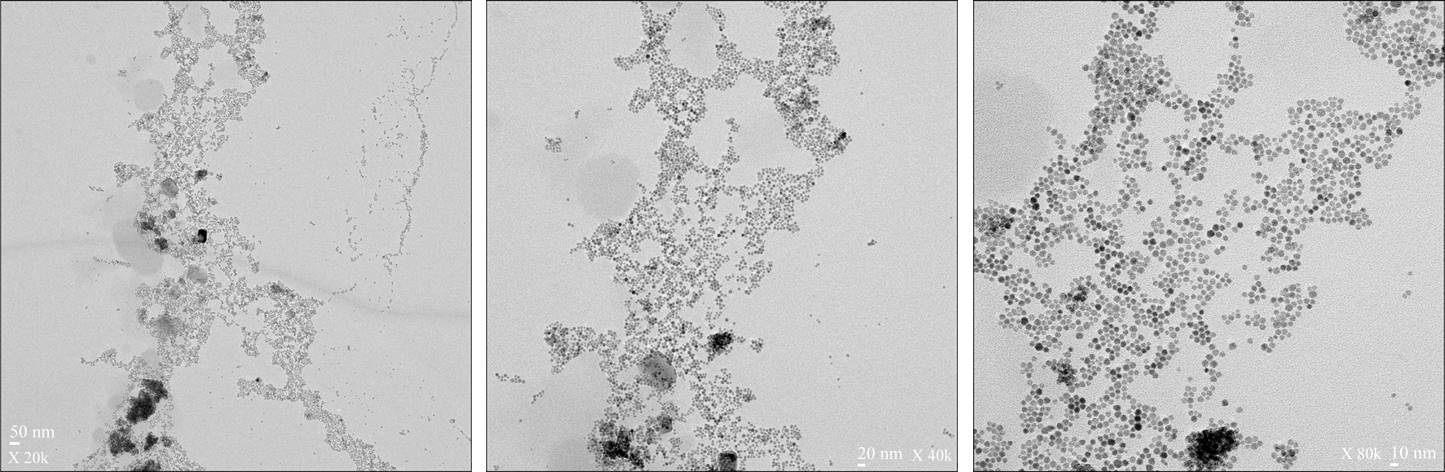


**NaGdF_4_:Eu**

**100 nm**

**a**

**Figure S1.** TEM micrographs of Eu (**a**) and Tb (**b**) doped NaGdF_4_ SciNPs. The SciNPs were dissolved in chloroform solution and dispensed on the Cu grid. **c**) TEM of NaGdF_4_:Tb SciNPs and the corresponding size distribution (**d**).


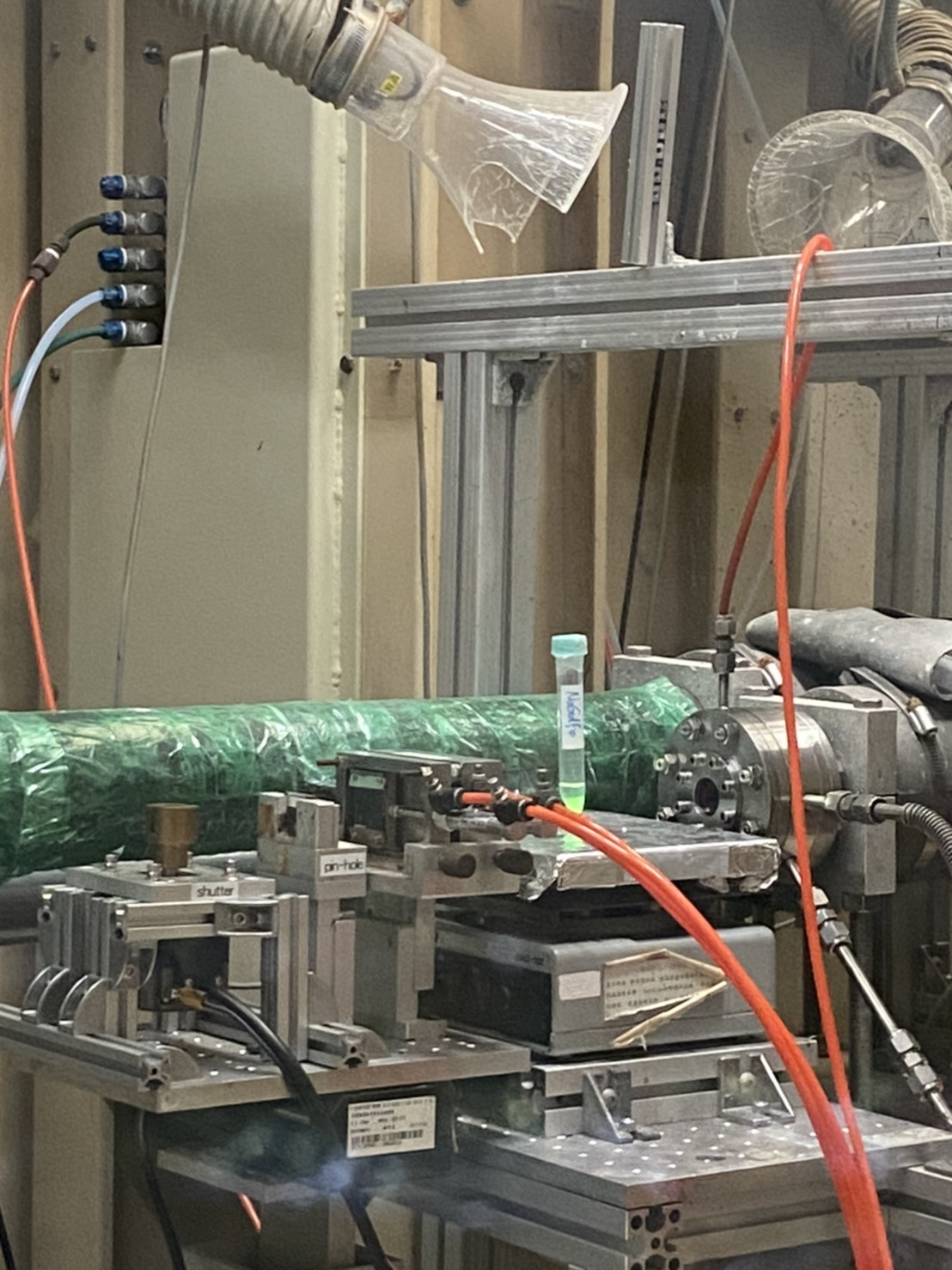

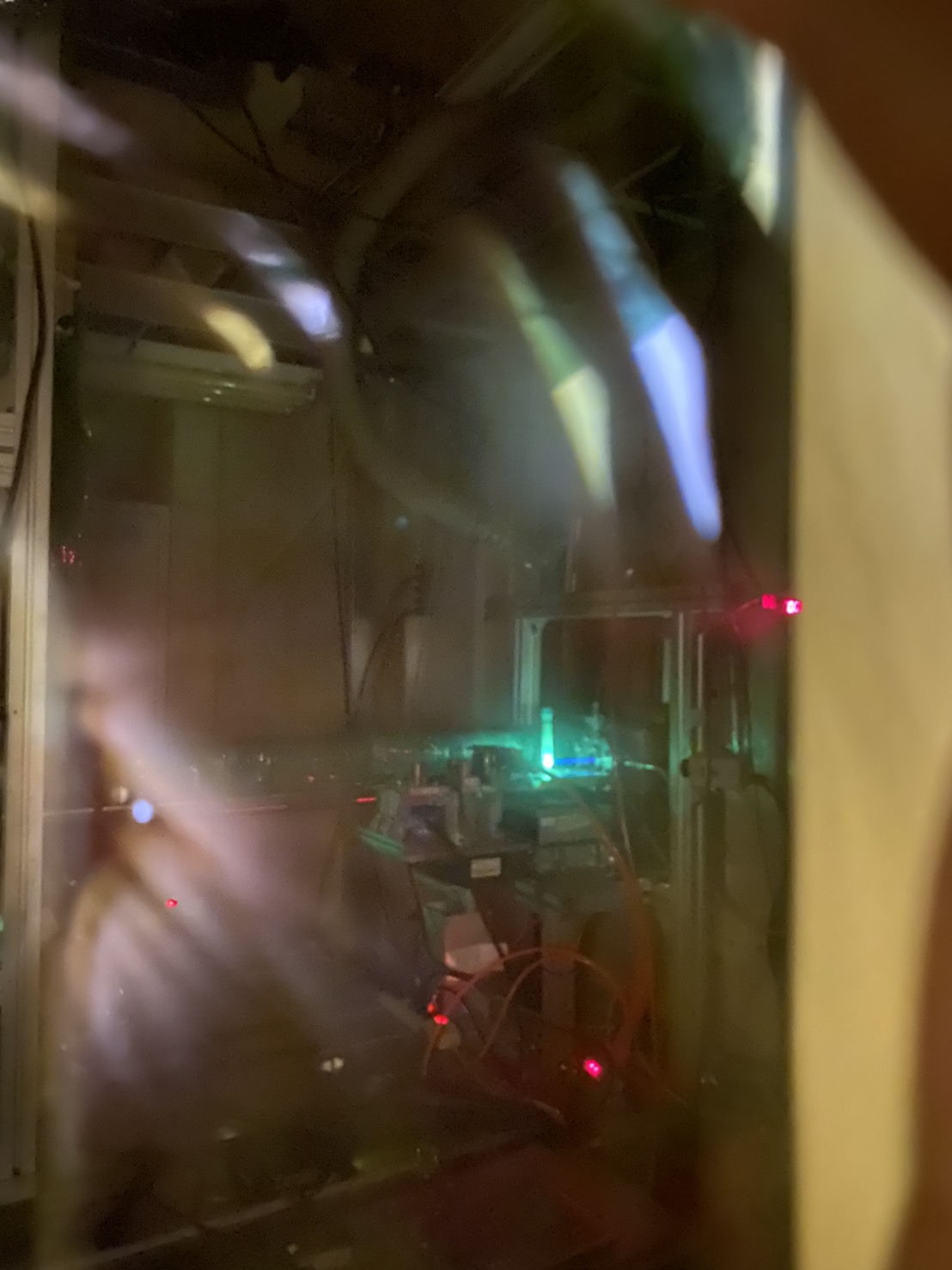

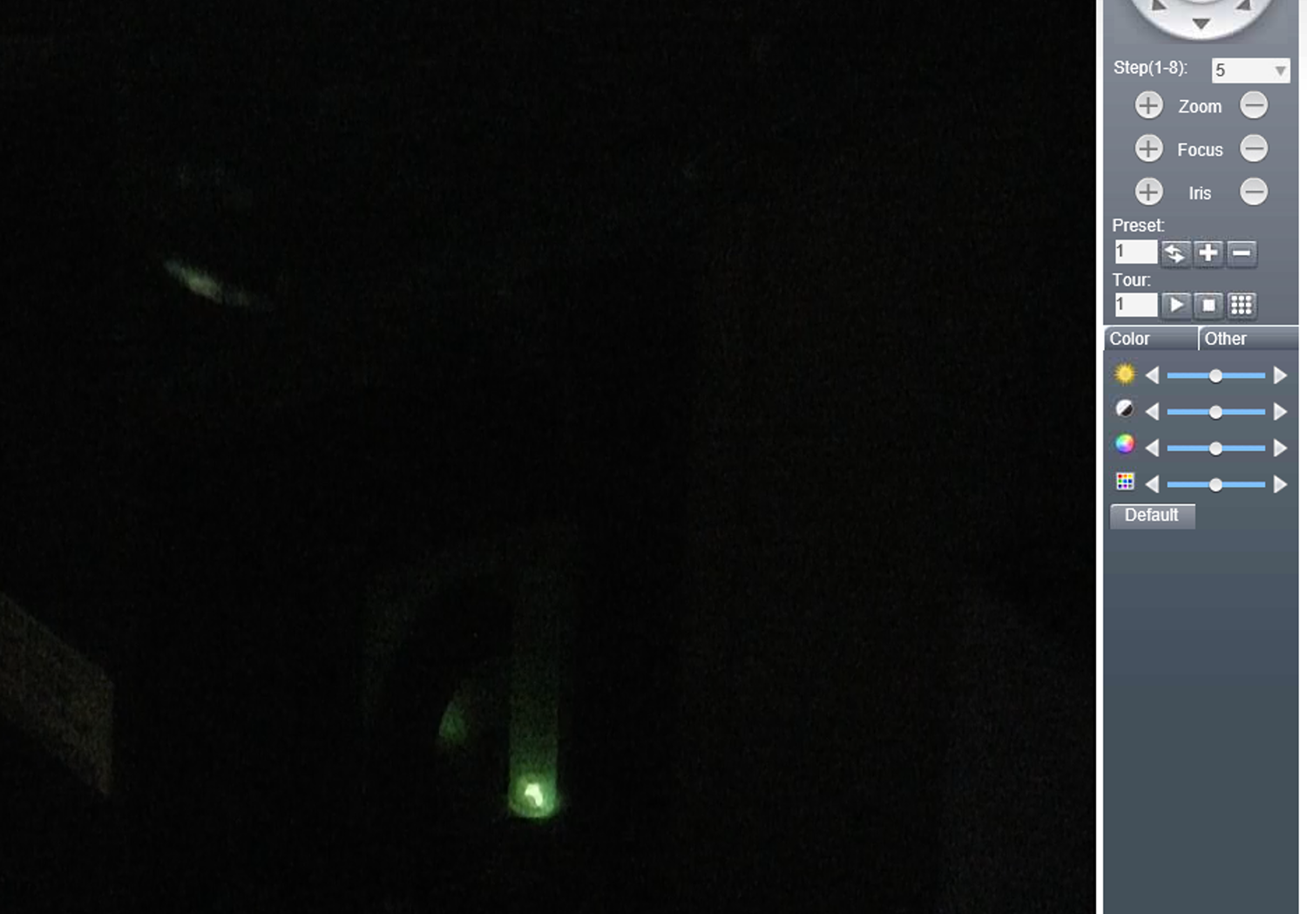

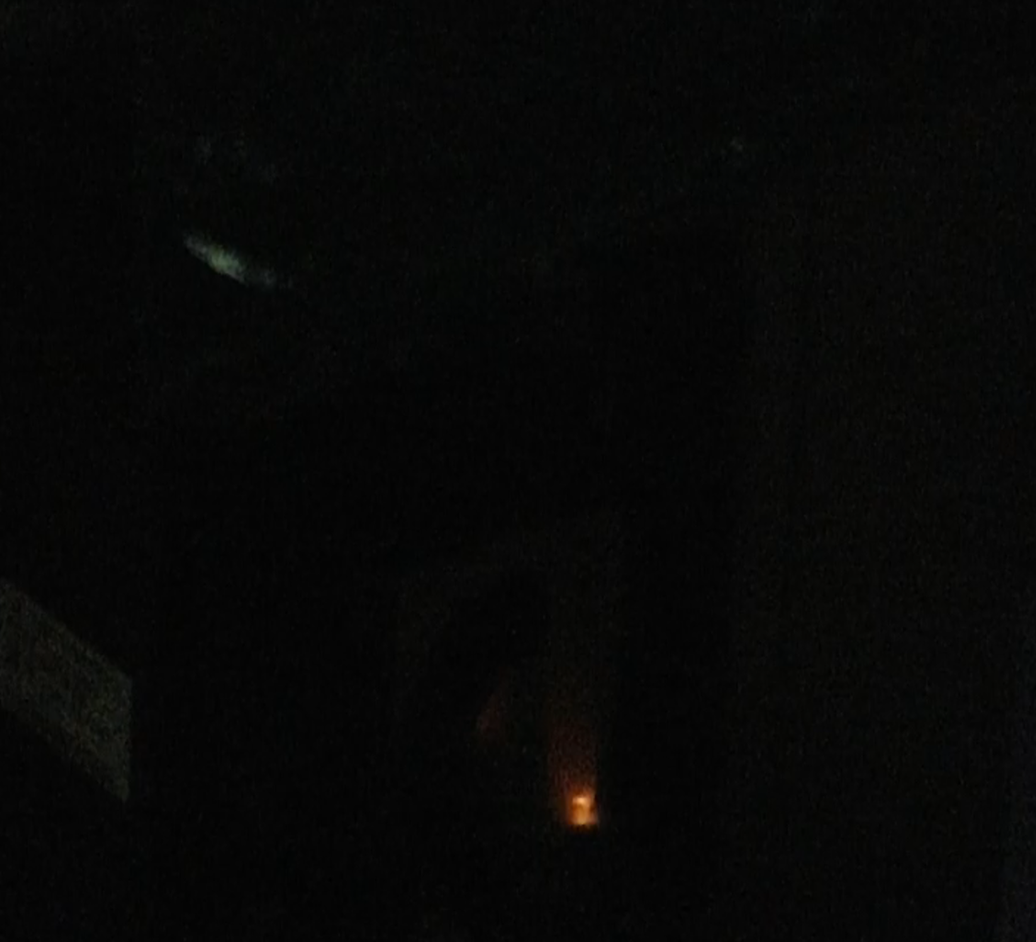


**NaGdF_4_:Tb**

**NaGdF_4_:Eu**

**Figure S2.** Pictures of NaGdF_4_ SciNPs emit different color visible light with Eu and Tb doping. A tube of chloroform solution containing NaGdF_4_: Tb SciNPs was placed under the synchrotron x-ray beam (left) and with x-ray irradiation (middle). The NaGdF_4_ SciNPs emit green (~540 nm) and red (610 nm) visible light with Tb and Eu doping (right).


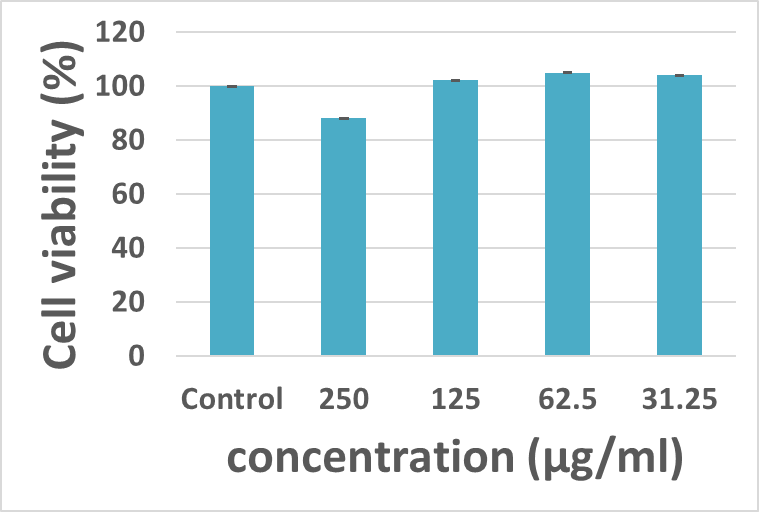


*p<0.05 compared with control

*

**Figure S3.** The cell viability test of NaGdF_4_: Eu SciNPs at different concentrations shows that the SciNPs are non-toxic. The cell survival rates (MTT assay) treated with different SciNPs concentrations shows only slight decrease to ~85% at the highest concentration.

**Video S02 (S02.mp4):**

**Video Title:** 3D reconstructed MAXWELL image of a Drosophila Larva.

**Video legends:** 3D reconstructed MAXWELL image of a Drosophila Larva fed with scintillating nanoparticles SciNPs (NaGdF_4_:Eu). The skeleton (blue) is visible due to x-ray autofluorescence.
